# Supplementary material for: Intervention effect of exercise on working memory in patients with depression: a systematic review
Source: PeerJ. 2024 Aug 28;12:e17986. doi: 10.7717/peerj.17986 (PMC11365484; doi:10.7717/peerj.17986)
Supplement: Supplemental Information 3 [file peerj-12-17986-s003.docx]

By musing on previous studies, it was found that the meta-analyses results of exercise improving working memory in patients with depression were also inconsistent. Sun et al. and Brondino et al.'s meta-analyses found no improvement in working memory in patients with depression after doing exercise(Brondino et al. 2017; Sun et al. 2018) . In contrast, Ren et al. and Contreras-Osorio et al.'s meta-analyses indicated that exercise can improve working memory in these patients(Contreras-Osorio et al. 2022; Ren et al. 2023). However, none of these studies explored the effects of exercise type, exercise intensity, exercise cycle, and intervention content on working memory, despite examining the impact of exercise on it. Therefore, this study aims to further investigate the intervention effects of exercise on working memory in patients with depression. Building upon previous researches, it seeks to clarify the dose-response relationships of various components of exercise on the intervention effects of working memory in an effort to identify the optimal exercise regimen, provide evidence-based recommendations for clinical practice and serve as a theoretical reference for researchers in the field.

Brondino N, Rocchetti M, Fusar-Poli L, Codrons E, Correale L, Vandoni M, Barbui C, and Politi P. 2017. A systematic review of cognitive effects of exercise in depression. *Acta Psychiatr Scand* 135:285-295. 10.1111/acps.12690

Contreras-Osorio F, Ramirez-Campillo R, Cerda-Vega E, Campos-Jara R, Martínez-Salazar C, Reigal RE, Hernández-Mendo A, Carneiro L, and Campos-Jara C. 2022. Effects of Physical Exercise on Executive Function in Adults with Depression: A Systematic Review and Meta-Analysis. *Int J Environ Res Public Health* 19. 10.3390/ijerph192215270

Ren FF, Alderman BL, Wang WG, Chen FT, Zhou WS, Zong WJ, Liang WM, and Chang YK. 2023. Effects of Exercise Training on Executive Functioning in Adults with Depression: A Systematic Review and Meta-Analysis of Randomized Controlled Trials. *Sports Med* 53:1765-1788. 10.1007/s40279-023-01869-2

Sun M, Lanctot K, Herrmann N, and Gallagher D. 2018. Exercise for Cognitive Symptoms in Depression: A Systematic Review of Interventional Studies. *Can J Psychiatry* 63:115-128. 10.1177/0706743717738493
